# In Planta Haploid Induction by Genome Editing of *DMP8* in *Pennisetum alopecuroides*

**DOI:** 10.1111/pbi.70789

**Published:** 2026-11-10

**Authors:** Yidi Guo, Xifeng Fan, Yuesen Yue, Lei Hua, Na Mu, Le Zhou, Lingyun Liu, Juying Wu, Ning Jia, Deying Li, Zhihui Chang, Ke Teng

**Affiliations:** ^1^ School of Grassland Science Beijing Forestry University Beijing China; ^2^ Institute of Grassland, Flowers and Ecology Beijing Academy of Agriculture and Forestry Sciences Beijing China; ^3^ Department of Plant Sciences University of Cambridge Cambridge UK; ^4^ College of Agricultural and Forestry Science and Technology Hebei North University Zhangjiakou China; ^5^ Department of Plant Sciences North Dakota State University Fargo North Dakota USA

1

Pennisetum Rich is an important C_4_ grass genus, which includes the world's sixth‐largest cereal crop and the highest‐yielding poaceous forage and bioenergy grass (Jin et al. 2025; Xu et al. 2024; Yan et al. 2023). However, its genetic improvement is still constrained by the long breeding cycles using conventional approaches such as successive inbreeding. Double haploid (DH) technology greatly accelerated the breeding process by fast production of homozygous lines through haploid induction and chromosome doubling (Zhong et al. 2019). Recent advances in in planta haploid induction have enabled efficient production of haploids in main crops (Liu et al. 2017; Zhong, Chen, et al. 2022; Zhong, Wang, et al. 2022). These systems rely on distinct genetic mechanisms, including loss‐of‐function of DOMAIN OF UNKNOWN FUNCTION 679 membrane protein (DMP) or phospholipase A (PLA1), and through egg cell expression of PARTHENOGENESIS(*PAR*) (Huang et al. 2024; Liu et al. 2017; Zhong et al. 2019).

*DMP* knocking‐out has been proved as a practicable strategy to induce haploids in both monocots and dicots through genome editing (Zhong et al. 2019; Zhong, Chen, et al. 2022; Zhong, Wang, et al. 2022). Nevertheless, *Pennisetum* species have historically posed challenges for genetic manipulation (Achary et al. 2025; Jin et al. 2025). Here, through CRISPR/Cas9‐mediated disruption of *PaDMP8*, we establish the first heritable in vivo haploid inducer in *Pennisetum alopecuroides* , a potential model plant and important breeding parents of *Pennisetum* with small genome, high in vitro regeneration and strong stress resistance, providing a key enabling technology for DH breeding in the genus of *Pennisetum* (Xu et al. 2024).

To identify the *DMP8* ortholog in *P. alopecuroides* , we screened the reference genome of ‘Liqiu’ (China Registered Cultivar Code:S‐BV‐PA‐007‐2021) using ZmDMP8 as query, this identified ten DMP homologues. Phylogenetic analysis grouped PaDMP8 with maize ZmDMP8 and *Arabidopsis thaliana* AtDMP8/AtDMP9, indicating PaDMP8 is the ortholog of ZmDMP8 (Figure S1). RT‐qPCR showed that *PaDMP8* was highly expressed in mature anthers but only weakly expressed in vegetative tissues, consistent with its role in the male gametophyte (Figure 1a). Subcellular localisation observation of both maize protoplasts and *Nicotiana benthamiana* epidermal cells confirmed PaDMP8 was localised to the plasma membrane (Figure 1b and Figure S2). Transgenic complementation assays demonstrated that *PaDMP8* restored the phenotype of the *A. thaliana* *dmp8dmp9* double mutant, supporting functional conservation of PaDMP8 (Figure 1c): *dmp8dmp9* mutant's seed set was 41.69%, while complementation lines 74 and 80 reached 68.63% and 76.59%, respectively, in agreement with *BnDMP1a* and *SlDMP* (Zhong, Chen, et al. 2022; Zhong, Wang, et al. 2022). To establish loss‐of‐function alleles, we designed sgRNAs targeting *PaDMP8* and deployed a CRISPR/Cas9 vector to carry out agrobacterium‐mediated transformation. Sanger sequencing of the target sites identified edits in 15 of 63 T_0_ plants (23.8%), and homozygous T_1_ lines carrying mutations at the target sites were subsequently uncovered and designated as *padmp8*‐2 and *padmp8*‐5 (Figure 1c).

**FIGURE 1:**
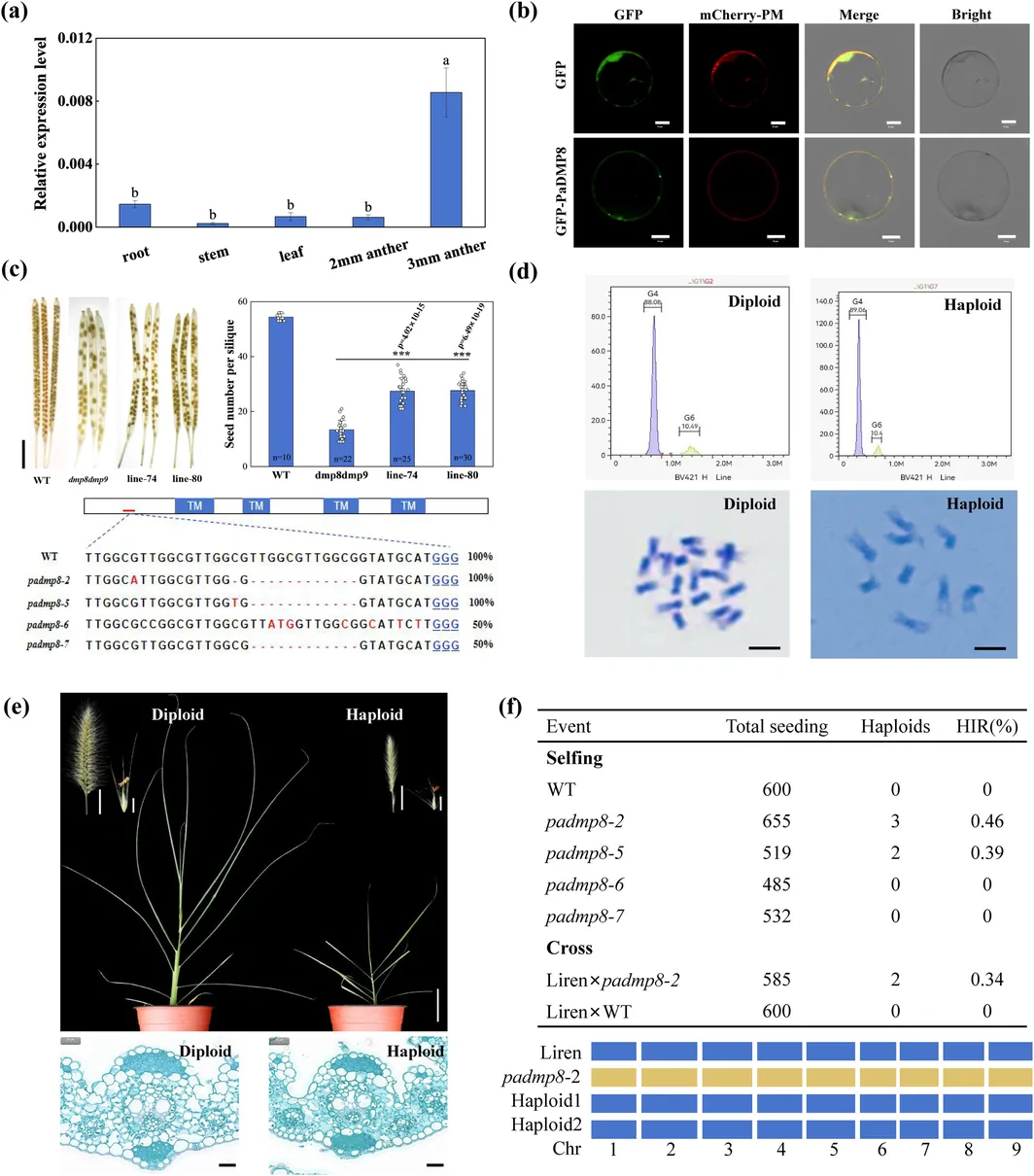
CRISPR/Cas9‐mediated knockout of *PaDMP8* triggers haploid induction in *P. alopecuroides* . (a) Tissue‐specific expression of *PaDMP8* (*n* = 4). (b) Subcellular localisation of PaDMP8. Bars, 10 μm. (c) Complementation of *A. thaliana* *dmp8dmp9* double mutants by overexpressing of *PaDMP8* and gene structure of *PaDMP8* and CRISPR/Cas9 editing results. Bar, 2 mm; blank boxes, coding regions; red lines, sgRNA target sites; blue boxes, TM domain. Data represent the mean ± SD; ***p < 0.001 (two‐tailed Student's *t*‐test); *n*, number of siliques. (d) Flow cytometry and chromosome counting results. Bars, 10 μm. (e) Phenotypic comparison between diploid and haploid plants. Bars: 5 cm for whole plants; 2 cm for spikes; 5 mm for flowers; 20 μm for leaf cross‐section. (f) Haploid induction rates (HIR) and SNP analysis of haploids and their parental plants.

To assess the haploid induction ability of *padmp8* mutants, we harvested their selfed progeny for screening haploids. Flow cytometry detection and chromosome squash observation of root tips identified 3 and 2 haploids in the self‐fertilised progeny of *padmp8‐2* and *padmp8‐5* respectively, corresponding to a haploid induction rate (HIR) of 0.46% (3/655) and 0.39% (2/519) (Figure 1d). Compared with diploid plants, haploids showed reduced growth vigour, including a 42% reduction in plant height, narrower and shorter leaves, defective inflorescences, and nearly complete male sterility (Figure 1e). Paraffin sections of leaves further showed that haploid plants had smaller and more densely packed cells, reduced cell length and decreased leaf thickness (Figure S3). We next used *padmp8‐2* as the pollen donor in crosses with *P. alopecuroides* ‘Liren’ (Registered Cultivar Code: Jing S‐SC‐PA‐024‐2017) and identified 2 haploids among 585 F1 progeny, giving an HIR of 0.34% (2/585) (Figure 1f). Whole genome sequencing results showed neither of the haploid seedlings carried paternally derived SNPs, confirming *padmp8* induced maternal haploids (Figure 1f). No haploids were detected in the selfed progeny of the wild type, excluding the possibility of spontaneous haploid formation under our experimental conditions (Figure 1f). Together, these findings demonstrate that CRISPR/Cas9‐mediated knockout of *PaDMP8* can successfully trigger in vivo maternal haploid induction in *P. alopecuroides* . The HIR achieved here (0.34%–0.46%) is similar to *ZmDMP8* (0.1%–0.3%) and *MtDMP8* knockout lines (0.29%–0.82%) (Wang et al. 2022; Zhong et al. 2019).

To our knowledge, we established the first in planta haploid induction system in the genus of *Pennisetum*, providing a key technical basis for genetic improvement and breeding in this important plant. Future work should focus on improving haploid induction efficiency by combining *PaDMP8* editing with additional haploid induction factors, such as homologues of *MTL/NLD/ZmPLA*, or by introducing egg cell ectopic expression of *PARs*. Such strategies will further accelerate the application of DH technology in *Pennisetum* breeding.

## Author Contributions

Ke Teng and Zhihui Chang designed the project. Yidi Guo, Na Mu, Le Zhou, and Lingyun Liu conceived the experiments. Yidi Guo and Ning Jia analysed data. Xifeng Fan, Yuesen Yue, and Juying Wu evaluated the phynotype. Yidi Guo, Lei Hua, and Deying Li wrote the manuscript with contributions from all authors.

## Funding

This work was supported by the National Natural Science Foundation of China (32573613), Scientific Funds of Beijing Academy of Agriculture and Forestry Sciences (KJCX20251011; KJCX20250917) and High‐End Foreign Experts Invitation Program (WZ2026‐06).

## Conflicts of Interest

The authors declare no conflicts of interest.

## Supporting information


**Figure S1:** Phylogenetic analysis of PaDMP8 with ZmDMP8 and AtDMP8/AtDMP9. Full‐length protein sequences were aligned using ClustalW, and a neighbour‐joining tree was constructed using MEGA6. Bootstrap percentages from 1000 replicates are indicated on branches; PaDMP8 is marked with a red star.
**Figure S2:** Subcellular localisation observation of PaDMP8 in *N. benthamiana* leaf epidermal cells. Bars: red, 50 μm; white, 25 μm.
**Figure S3:** Paraffin sections of diploid and haploid plants leaves. Longitudinal sections show reduced cell size and compact arrangement in haploids compared to diploids. Bars, 20 μm.

**Table S1:** Primers used in this study.

## Data Availability

The data that support the findings of this study are available on request from the corresponding author. The data are not publicly available due to privacy or ethical restrictions.
