# Supplementary material for: In Planta Haploid Induction by Genome Editing of DMP8 in Pennisetum alopecuroides
Source: Plant Biotechnol J. 2026 Nov 10:10.1111/pbi.70789. Online ahead of print. doi: 10.1111/pbi.70789 (PMC13749013; doi:10.1111/pbi.70789)
Supplement: Supplementary file 1 [file PBI-9999-0-s001.docx]

**Supplemental material**

*In planta* haploid induction by genome editing of *DMP8* in *Pennisetum* *alopecuroides*

Yidi Guo^1,2^, Xifeng Fan^2^, Yuesen Yue^2^, Lei Hua^3^, Na Mu^2^, Le Zhou^2^, Lingyun Liu^2^, Juying Wu^2^, Ning Jia^4^, Deying Li^5^, Zhihui Chang^1^*, Ke Teng^2^*

1. School of Grassland Science, Beijing Forestry University, Beijing, 100083, China

2. Institute of Grassland, Flowers and Ecology, Beijing Academy of Agriculture and Forestry Sciences, Beijing, 100097, China

3. Department of Plant Sciences, University of Cambridge, Cambridge, CB2 3EA, United Kingdom

4. College of Agricultural and Forestry Science and Technology, Hebei North University, Zhangjiakou, 075131, China

5. Department of Plant Sciences North Dakota State University, Fargo, ND 58108, United States

*Correspondence: changzh@bjfu.edu.cn, tengke@baafs.net.cn

**Supplemental methods**

**Plant materials and growth conditions**

*Pennisetum alopecuroides* cv. ‘Liqiu’ (China Registered Cultivar Code:S-BV-PA-007-2021) and ‘Liren’ (Registered Cultivar Code: Jing S-SC-PA-024-2017) was used in this study. It was grown in a well-conditioned greenhouse at Beijing Academy of Agriculture and Forestry Sciences. *Arabidopsis thaliana* ecotype ‘Columbia’, *dmp8dmp9* double mutant line were cultivated in an RXZ-380D-LED growth chamber (Ningbo Jiang Nan Instrument Factory, Ningbo, China) set at 24/22℃ with 65% humidity and approximately 400 μmol m^−2^S^-1^ PAR. *Nicotiana benthamiana* seedlings and *Zea mays* cv. ‘ZD958’ were planted in another growth chamber set at 28/25℃ with 65% humidity and approximately 400 μmol m^−2^S^-1^ PAR.

**Identification and expression analysis of *PaDMP8***

The protein sequence of maize DMP8 was used as a query to identify homologous proteins against the reference genome of *P. alopecuroides* using TBtools-II (Chen et al.,2023). Total RNA was extracted from leaves of ‘Liqiu’ using the FastPure Universal Plant Total RNA Isolation Kit (Vazyme, Nanjing, China). First-strand cDNA was synthesized using PrimeScript RT Master Mix (Takara, Dalian, China). The open reading frame of *PaDMP8* was amplified using the cDNA as template with the gene-specific primers PaDMP-F/R. The PCR product was purified and inserted into the pMD19-T vector, and then transformed into DH5α competent cells. Positive clones were confirmed by colony PCR and Sanger sequencing and subsequently used for vector construction. Quantitative real-time PCR was conducted on a Bio-Rad CFX system using TB Green Premix Ex Taq (Takara, Dalian, China) with *PaActin* as housekeeping gene. Relative expression levels were calculated using the ΔΔCt method (Teng et al., 2021). The primers were listed in supplementary Table S1.

**Subcellular localization observation**

The coding sequence of *PaDMP8* without the stop codon was cloned into pUC19-35S-EGFP and pCambia3302Y3 to generate the GFP-PaDMP8 or PaDMP8-YFP fusion constructs, respectively. The GFP-PaDMP8 vector was transformed in maize protoplasts according to the method described by Zhong et al. (2019). The PaDMP8-YFP plasmid was transformed into *Agrobacterium tumefaciens* strain EHA105 and transiently expressed in leaves of 21-day-old *N. benthamiana* plants by *Agrobacterium*-mediated infiltration (Teng et al., 2016). Finally, GFP and YFP fluorescence signals were observed using a Leica SP8 laser confocal scanning microscope after 16h or 48 h, respectively.

**Functional complementation assay in *A. thaliana***

The *PaDMP8* coding sequence was cloned into pCambia3302Y3 expression vector. The plasmid was transformed into the *A. thaliana dmp8dmp9* double mutant using the floral dip method (Jia et al., 2016). Transgenic seedlings were screened using 60 mg L^-1^ glufosinate. T_3_ generation was utilized to record the silique morphology and seed-setting rate.

**CRISPR/Cas9 vector construction and genetic transformation of *P. alopecuroides***

Target sites of *PaDMP8* were designed using the CRISPOR online tool. Then tRNA-sgRNA constructs was assembled into the pYLCRISPR/Cas9Pubi-H backbone according to a previously reported protocol (Yang et al., 2025). The plasmid was introduced into *A. tumefaciens* strain GV3101 and used for stable transformation of *P. alopecuroides* cv. ‘Liqiu’. Callus was induced from immature inflorescences of ‘Liqiu’ and were sub-cultured twice before infection. Pre-cultured callus were infected with *Agrobacterium* suspension at an OD_600_ of 0.4–0.6 and subsequently subjected to selection, differentiation and rooting medium to obtain regenerated plants.

**Generation of inducer line and haploid screening**

Genomic DNA was extracted from regenerated plants using CTAB method. Transgenic plants were first identified by PCR using the primers Cas9-F/R. The target region of *PaDMP8* was then amplified using the flanking primers seqDMP-F/R and subjected to Sanger sequencing. Mutation types were analyzed using DNAMAN 8.0. Mutant lines were selected and used to induce haploid via selfing and crossing. With ‘Liqiu’ as diploid control, ploidy levels of the progeny seedlings were determined by flow cytometry, and chromosomes were photographed and calculated via the root-tip chromosome squash method. The phenotype of the identified haploids was recorded by a Cannon EOS 80D digital camera. The cell morphology of leaves was observed using paraffin sectioning.

**Whole-genome resequencing and genotype calling**

This section was carried out based on the well-established method with appropriate modifications (Wang et al., 2019). Briefly, the 150-bp paired-end reads were generated by DNBSEQ-T7 at an average depth of 20-fold coverage for each sample. Clean reads were filtered from raw reads using fastp with default parameters. The clean reads were then aligned against to the *P. alopecuroides* reference genome using BWA-MEM2. GATK HaplotypeCaller was used to generate high-quality SNPs with parameters “--emit-ref-confidence GVCF --heterozygosity 0.05 --indel-heterozygosity 1.25E-4 --standard-min-confidence-threshold-for-calling 30.0 --minimum-mapping-quality 20 --min-base-quality-score 10”. GATK GenotypeGVCFs was then utilized to generate the joint genotyping of all samples.

**Material and data availability**

For research purpose, the plasmids, inducer lines, haploid lines, as well as raw data are available from Z.C and K.T upon request.

**Supplemental references**

Chen C, Wu Y, Li J, Wang X, Zeng Z, Xu J, Liu Y, Feng J, Chen H, He Y and Xia R (2023) TBtools-II: A “one for all, all for one” bioinformatics platform for biological big-data mining. *Molecular Plant* 16:1733-1742.

Jia N, Liu X and Gao H (2016) A DNA2 Homolog is required for DNA damage repair, cell cycle regulation, and meristem maintenance in plants. *Plant Physiology* 171:318-333.

Teng K, Tan P, Guan J, Dong D, Liu L, Guo Y, Guo W, Yue Y, Fan X and Wu J (2021) Functional characterization of the chlorophyll b reductase gene NYC1 associated with chlorophyll degradation and photosynthesis in Zoysia japonica. *Environmental and Experimental Botany* 191:104607.

Teng K, Tan P, Xiao G, Han L, Chang Z and Chao Y (2016) Heterologous expression of a novel Zoysia japonica salt-induced glycine-rich RNA-binding protein gene, ZjGRP , caused salt sensitivity in Arabidopsis. *Plant Cell Reports* 36:1-13.

Yang H, Yao J, Hao H, Jiang L, Li J, Chi Y, Zhang J and Xu B (2025) Promoter editing of LpNOL generates stay-green perennial ryegrass with improved forage quality and heat tolerance. *Plant Physiology* 199:kiaf402.

Zhong Y, Liu C, Qi X, Jiao Y, Wang D, Wang Y, Liu Z, Chen C, Chen B, Tian X, Li J, Chen M, Dong X, Xu X, Li L, Li W, Liu W, Jin W, Lai J and Chen S (2019) Mutation of ZmDMP enhances haploid induction in maize. *Nature Plants* 5:575-580.

Wang C, Liu Q, Shen Y, Hua Y, Wang J, Lin J, Wu M, Sun T, Cheng Z, Mercier R, Wang K. (2019). Clonal seeds from hybrid rice by simultaneous genome engineering of meiosis and fertilization genes. *Nature Biotechnology* 37: 283–286.

**Supplemental figures**

**Figure S1.** Phylogenetic analysis of PaDMP8 with ZmDMP8 and AtDMP8/AtDMP9. Full-length protein sequences were aligned using ClustalW, and a neighbor-joining tree was constructed using MEGA6. Bootstrap percentages from 1,000 replicates are indicated on branches; PaDMP8 is marked with a red star.

**Figure S2.** Subcellular localization observation of PaDMP8 in *N. benthamiana* leaf epidermal cells. Bars: red, 50 μm; white, 25 μm.

**Figure S3.** Paraffin sections of diploid and haploid plants leaves. Longitudinal sections show reduced cell size and compact arrangement in haploids compared to diploids. Bars, 20 μm.
