# Supplementary material for: In Planta Haploid Induction by Genome Editing of DMP8 in Pennisetum alopecuroides
Source: Plant Biotechnol J. 2026 Nov 10:10.1111/pbi.70789. Online ahead of print. doi: 10.1111/pbi.70789 (PMC13749013; doi:10.1111/pbi.70789)
Supplement: Supplementary file 2 [file PBI-9999-0-s002.docx]

Table S1 Primers used in this study

| Primer name | Sequence (5’-3’) | Usage |
| --- | --- | --- |
| PaDMP-F | TTGTTATGCGTAGAAGCAGCCG | gene clone |
| PaDMP-R | GCAATTTGGAACGGATGGAGTA | gene clone |
| qPaDMP-F | GGTACCGGCTCGCCTTCATC | RT-qPCR |
| qPaDMP-R | TCACCTTCCCCATCTCCTCG | RT-qPCR |
| PaActin-F | CATTGAACCCACTGACACGA | RT-qPCR |
| PaActin-R | AGGACAAGATGAAGGACGGA | RT-qPCR |
| 3302Y3-PaDMP-F | cctactagtcctagggacgtcaATGGCGGGCATTCGAACTCC | subcellular localization |
| 3302Y3-PaDMP-R | tgctcaccatacgcgttacagaAACTGCCATAGTCTGGTCTG | subcellular localization |
| pUC19-35S-EGFP-F | tggacgagctgtacaagggtacccccgggATGGCGGGCATTCGAACTCC | subcellular localization |
| pUC19-35S-EGFP-R | accgatgatacgaacgaaagctctgcagTTAAACTGCCATAGTCTGGTCTG | subcellular localization |
| pYL-DMP-F2 | *ttggcgttggcggtatgcAT*GTTTTAGAGCTAGAAATAGCAAG | CRISPR/Cas |
| pYL-DMP-R1 | *atgcataccgccaacgccaa*TGCACCAGCCGG | CRISPR/Cas |
| pYL-DMP-R3 | *ctacatagcccagcaaggca*TGCACCAGCCGG | CRISPR/Cas |
| pYL-DMP-F4 | *tgccttgctgggctatgtag*GTTTTAGAGCTAGAAATAGCAAG | CRISPR/Cas |
| pYL-DMP-R5 | TTCAGAGGTCTCA*gaacgtcgcgag*TGCACCAGCCGG | CRISPR/Cas |
| pYL-DMP-F6 | GAGGTCTCG*gttcccgaggag*GTTTTAGAGCTAGAAATAGCAAG | CRISPR/Cas |
| seqPaDMP-F | CATTCGAACTCCGAACCACC | screening of mutation |
| seqPaDMP-R | TGCCCGTGGGGAGGAAGTTG | screening of mutation |
| Hyg-F | TCGTTATGTTTATCGGCACTTTG | transgenic verification |
| Hyg-R | CCCGCTCGTCTGGCTAAGAT | transgenic verification |
| Cas9-F | GCTACGCCGGTTACATTGAC | transgenic verification |
| Cas9-R | GCTCGTTGTAGACCGTGAAGTA | transgenic verification |
